# The Five-Year Growth of an Orthopedic Urgent Care Center: Identifying Patient and Center Trends

**DOI:** 10.7759/cureus.32176

**Published:** 2022-12-04

**Authors:** Tyler W Henry, Kevin Lutsky, Pedro Beredjiklian, Jonas Matzon

**Affiliations:** 1 Orthopedic Surgery, Rothman Orthopaedic Institute, Philadelphia, USA; 2 Orthopedic Surgery, University of Vermont, Burlington, USA; 3 Hand Surgery, Rothman Orthopedic Institute, Philadelphia, USA

**Keywords:** injuries, practice management, patient trends, urgent care, orthopaedic

## Abstract

Introduction: With emergency department utilization rising at a dramatic rate, orthopedic urgent care centers (oUCCs) have become increasingly popular. The financial viability and basic advantages of oUCCs have been described in the literature, but little is known about the characteristics of patients treated and the diagnoses encountered. The purpose of this study is to report and evaluate the patients and diagnoses that are most commonly seen in an oUCC so that future care may be better tailored to the needs of the patients seeking these services.

Materials and methods: All patients seen at a single suburban oUCC in its first and fifth years of operation (2014 and 2019) were identified. The medical records were reviewed to assess patient demographics, diagnoses encountered, and services rendered. The clinical courses of patients treated were also reviewed to identify those who underwent eventual surgery for their presenting complaint.

Results: A total of 24,756 patient visits occurred during the study period, and the number of visits nearly doubled between the first and fifth years (8,301 in 2014 and 16,455 in 2019). The most common diagnoses encountered were lower leg pain, back pain, and foot/ankle pain. Radiographs were obtained in 17,236 visits (70%), most commonly of the knee, elbow, foot, or ankle. A total of 1,334 patients (5.4%) underwent eventual surgery for their presenting complaint - defined as a surgical conversion. Of all the orthopedic subspecialties, sports medicine had the highest rate of surgical conversion (29% of all conversions). The surgical conversion rate increased slightly from year one (4.7%) to year five (5.8%).

Conclusions: OUCCs are an effective means of expanding access to care for patients and increasing the volume of an orthopedic practice. Continued monitoring of the types of patients seen within oUCCs will further optimize care delivery.

## Introduction

Providing timely access to care in a resource-conscious manner remains a priority for physicians regardless of specialty. With emergency department (ED) utilization rising at a dramatic rate [1], the implementation of urgent care centers (UCCs) has bridged the gap between scheduled outpatient appointments and immediate ED visits to better address patient demand [2]. UCCs allow for expanded availability compared to traditional clinical visits and for decreased waiting times compared to many EDs [3]. As the popularity of UCCs continues to grow [4], specialty-specific UCCs have become the next evolution to more closely meet the needs of patients seeking care. Orthopedics is a specialty of particular interest when considering the value of a dedicated UCC, as approximately 15% of all ED visits are for musculoskeletal complaints [5]. In fact, dedicated orthopedic urgent care centers (oUCCs) operated by orthopedic practices have become increasingly common, and existing reports suggest they can serve as an integral component within the broader healthcare system [3].

The financial viability and basic advantages of oUCCs (decreased wait times and decreased ED burden) have previously been described in the literature [3]. However, it is presently unclear what orthopedic conditions patients present with to these oUCCs and whether these conditions commonly necessitate surgery. Furthermore, understanding how patients are using oUCCs provides valuable insight into their clinical utility for continued implementation. We, therefore, sought to describe the patient visits at a suburban oUCC in the northeastern United States during its first and fifth years of operation (2014 and 2019). We secondarily compared the differences between visits in years one and five to assess the evolution of care provided at the oUCC. The purpose of this study was to determine the patients and diagnoses that are most commonly encountered in an oUCC.

## Materials and methods

Following Institutional Review Board approval (Thomas Jefferson University Control #13D.432), with a waiver of informed consent per institutional protocol, a database search was conducted to identify all patient visits at a single suburban oUCC in 2014 and 2019. The oUCC opened in the fall of 2013, with 2014 serving as its first full year in operation. The center is located approximately 15 miles from a major city in the northeastern United States and is operated by a multi-specialty regional orthopedic practice, which has an outpatient clinic within the same facility as the oUCC. During the study period, patient visits were conducted seven days a week from 8 AM to 8 PM on weekdays and from 9 AM to 6 PM on weekends. Covering providers included orthopedic surgeons, non-operative sports medicine physicians, orthopedic physiatrists, and orthopedic physician assistants. Patients who were seen in the oUCC were referred by the treating provider for outpatient follow-up as warranted by the presenting complaint/condition.

Patient demographics, diagnosis codes, and treatment provided (i.e., radiographic evaluation, splinting, injection, etc.) were collected for every visit. During the first year of operation (2014), diagnosis codes were assigned using the International Classification of Diseases, Ninth Revision (ICD-9), but by the fifth year of operation (2019), coding was performed using ICD-10. Patients who were directly transferred to an ED for immediate, higher-level treatment were identified. The clinical course of all patients was tracked. All patients who underwent surgery at our institution related to their presenting complaint within 9 months of the initial oUCC visit were recorded and labeled as surgical conversions.

All data were compiled and analyzed using the Statistical Package for the Social Sciences (SPSS Inc., Ver 26.0, Chicago, IL). Descriptive statistics were used to report all patient and visit characteristics. Chi-Square analysis was used to compare rates of surgical conversions, with the level of significance established at p < 0.05.

## Results

A total of 24,756 visits occurred during the study period, and the number of visits nearly doubled between the first and fifth years (8,301 in 2014 and 16,455 in 2019). The age of patients seen ranged from 3 to 101 years old, with the 11-17 age range most common in the first year (20%) and 65+ most common in year five (22%) (Tables 1, 2). A total of 5,135 visits (20.8%) involved patients under the age of 18. Most patients seen were female (56%).

**Table 1:** Breakdown of patient age ranges for visits occurring in the first year of operation.

Age Range	Total	Percentage
3-10	214	3%
11-17	1,640	20%
18-24	630	8%
25-34	727	9%
35-44	1,031	12%
45-54	1,460	18%
55-64	1,194	14%
65+	1,405	17%
Total	8,301	

**Table 2:** Breakdown of patient age ranges for visits occurring in the fifth year of operation.

Age Range	Total	Percentage
3-10	462	3%
11-17	2,837	17%
18-24	916	6%
25-34	1,073	7%
35-44	1,924	12%
45-54	2,871	17%
55-64	2,779	17%
65+	3,593	22%
Total	16,455	

The most common diagnoses encountered were lower leg pain, back pain, and foot/ankle pain (Tables 3, 4). In year 1, six (30%) of the top 20 most common diagnoses were related to the lower leg/ankle/foot, four (20%) were related to the spine, three (15%) were related to the knee, and two (10%) were related to the shoulder, hand/wrist, and hip/thigh respectively. In year 5, five (25%) of the top 20 diagnoses were related to the foot/ankle, three (15%) were related to the knee, spine, and hand/wrist respectively, and two (10%) were related to the shoulder, elbow and hip. Radiographs were obtained in 17,236 visits (70%).

**Table 3:** The most common primary diagnoses encountered during the first year of operation. *Initial ICD-9 code converted to closest equivalent ICD-10 code. ICD = International Classification of Diseases

Top 20 Diagnoses		
Rank	ICD Code*	Description
1	M25.569	Pain in joint, lower leg
2	M17.10	Osteoarthrosis, localized, primary, lower leg
3	S93.429A	Sprain of ankle, unspecified site
4	M54.5	Low back pain
5	M54.14	Thoracic or lumbosacral neuritis or radiculitis, unspecified
6	M25.539	Pain in joint, forearm
7	M25.579	Pain in joint, ankle and foot
8	M25.559	Pain in joint, pelvic region and thigh
9	M75.100	Disorders of bursae and tendons in shoulder region, unspecified
10	M22.40	Chondromalacia of patella
11	M79.609	Pain in limb
12	M54.2	Cervicalgia
13	S83.249A	Tear of medial cartilage or meniscus of knee, current
14	M48.061	Spinal stenosis, lumbar region, without neurogenic claudication
15	S83.90XA	Sprains and strains of unspecified site of knee and leg
16	M16.10	Osteoarthrosis, localized, primary, pelvic region and thigh
17	G56.00	Carpal tunnel syndrome
18	S93.609A	Sprain of foot, unspecified site
19	M25.519	Pain in joint, shoulder region
20	M65.879	Tenosynovitis of foot and ankle

**Table 4:** The most common primary diagnoses encountered during the fifth year of operation. ICD = International Classification of Diseases

Top 20 Diagnoses		
Rank	ICD Code	Description
1	M25.561	Pain in right knee
2	M25.562	Pain in left knee
3	M54.5	Low back pain
4	M79.672	Left foot pain
5	M79.671	Right foot pain
6	M54.2	Cervicalgia
7	M25.571	Acute right ankle pain
8	M54.16	Radiculopathy, lumbar region
9	M25.572	Acute left ankle pain
10	M25.511	Pain in right shoulder
11	M17.12	Unilateral primary osteoarthritis, left knee
12	M25.551	Pain in right hip
13	M25.512	Pain in left shoulder
14	M25.532	Arthralgia of left wrist
15	M25.552	Acute pain of left hip
16	M25.531	Pain in right wrist
17	M79.644	Pain in right finger(s)
18	S93.402A	Sprain of unspecified ligament of left ankle, initial encounter
19	M25.521	Arthralgia of elbow, right
20	M25.522	Arthralgia of elbow, left

A total of five patients (0.06% of 2014 visits) were directly transferred from the oUCC to a nearby ED for further management during the first year of operation, which was the only year this statistic was directly tracked. A total of 1,334 patients (5.4%) eventually underwent surgery by our practice for their presenting complaint. Sports medicine was the most common subspecialty-related pathology for which surgical intervention was required (29% of surgeries) (Table 5). The overall surgical conversion rate increased slightly from year 1 (4.7%) to year 5 (5.8%) (p < 0.01).

**Table 5:** Converted surgeries from oUCC visits in the first and fifth years of operation. *Division represents the division within the orthopedic practice to which the operative surgeon belongs. The divisional organization may differ between orthopedic practices. oUCC = Orthopedic Urgent Care Center

Division*	Surgical Conversions		% of Total Surgeries Scheduled	
	Year One	Year Five	Year One	Year Five
Sports Medicine	144	240	37%	25%
Hand & Wrist	91	197	24%	21%
Joint Replacement	39	185	10%	20%
Spine	16	131	4%	14%
Foot & Ankle	56	96	14%	10%
Shoulder & Elbow	39	82	10%	9%
Oncology	1	6	0.3%	0.6%
Trauma	1	6	0.3%	0.6%
Generalist	0	4	0%	0.4%
	Total = 387	Total = 947		

In both years, the months with the highest numbers of visits were August (2,375 total visits), July (2,323 total visits), October (2,129 total visits), and September (2,082 total visits). Monday was the busiest day of the week (4,763 total visits), while Sunday had the fewest visits (2,650 total visits). The majority of visits (8,142 total, 33%) were between the hours of 9 AM and 12 PM. In terms of the geographical catchment area, most patients (67%) resided within 20 miles of the oUCC.

## Discussion

OUCCs have proven to be a valuable addition to the healthcare system by increasing access to care and decreasing the ED burden [3]. Our oUCC demonstrated tremendous growth in the first five years of operation serving a wide range of patients within the catchment area. Back pain and acute lower extremity injuries were the most common diagnoses, and patients usually required radiographic evaluation. About 5% of encounters resulted in surgical conversions, and this percentage increased slightly over time. Given that the vast majority of visits did not require surgical treatment, the oUCC may have helped to limit the clinical burden for surgeons within the practice by properly triaging and diverting non-surgical pathologies to more appropriate care providers. At the same time, patients still had the benefit of being seen and evaluated by a specialist and were able to have their care directed by someone with a potentially higher level of clinical expertise in their condition than if they had been evaluated by a non-orthopedic provider at their primary care office or non-specialty UCC or ED.

The financial viability of oUCCs was previously reported by Anderson and Althausen through a review of 12,722 OUCC visits across 12 months [3]. The total revenue generated from these visits alone was just below $1.7 million and follow-up care generated an additional $7.7 million for the orthopedic practice [3]. Furthermore, the diversion of care from local EDs to the oUCC resulted in a healthcare system savings of over $97 million while also saving patients nearly two hours per episode of care [3]. Limiting patient volume within EDs had even more importance during the COVID-19 pandemic, which triggered a shift in ED utilization amid substantially higher concerns for viral exposure to patients who are otherwise healthy. This particular utility was previously quantified by MacKechnie et al., who found that the implementation of an orthopedic walk-in clinic decreased ED referrals by 40.6% [6]. Adding these advantages to the two-fold growth demonstrated across five years in our oUCC underscores the value of oUCC implementation to the patient, the orthopedic practice, and the healthcare system as a whole.

We attribute the majority of the experienced growth between years 1 and 5 to local marketing efforts, continued practice expansion within the catchment area, and growing informal referral sources throughout this period (i.e., former patients). With the financial viability and growth potential supported, it is important to next consider how best to optimize care through appropriate staffing of the oUCC. A myriad of factors goes into the staffing of oUCCs, but the present findings and existing literature suggest that non-operative physicians and physician extenders can deliver the necessary scope of care that most presenting patients require [7]. Physician extenders and non-operative physicians were involved in a large portion of the visits to our OUCC during the study period. The small percentage of visits necessitating immediate ED transfers within the first year of operation suggests that the pathologies encountered were well within the scope and expertise of these providers and that the vast majority of acute care could be provided within an oUCC setting. Aside from the acuity of diagnoses, oUCCs must consider the variety of patients that may present. For instance, pediatric patients represented about 20% of the total population treated. It, therefore, befits the oUCC to ensure providers comfortable with treating children are readily available at all times.

Late summer and early fall were the busiest months within the oUCC. Prior studies have demonstrated seasonal variation in upper extremity orthopedic diagnoses [8]. With respect to the oUCC, we suspect this correlates with an uptick in outdoor recreational activity in the area during these times of the year. Overall, Monday mornings were the busiest periods within our oUCC over both years, which we hypothesize is a byproduct of injuries occurring over the weekend. It seems logical that this daily usage pattern would hold true regardless of geography, while it is likely that regional weather, population, or tourism differences could substantially impact seasonal usage trends. oUCC implementation should certainly incorporate such factors when determining staffing needs and actively track usage to adjust accordingly.

Prior reports in the literature have also described the value of orthopedic triage models properly stratifying surgical and non-surgical care to more efficiently and effectively treating patients. In a study of 2,651 patients first seen by an extended scope practitioner (physiotherapists practicing in the United Kingdom), Wood et al. reported that only 8% of patients required surgical consultation, and those not requiring escalation of care were treated with great outcomes and high patient satisfaction [9]. Although the practice models differ, a similar benefit was seen in our study population. The approximately 95% of patients seen in our oUCC who did not require surgical intervention represent over 23,000 patients who would have otherwise waited some period to schedule a clinical visit or gone directly to a nearby ED. From year one to year five, the number of physician extenders covering oUCC shifts increased, and importantly, this was accompanied by an increase in surgical conversion rates. It appears clear that non-operative providers can triage patients within an oUCC and appropriately refer those requiring surgery. However, if surgeon coverage is desired within an oUCC, it is worth considering which surgical pathologies are commonly encountered. Within our oUCC and practice, these were most often soft tissue injuries treated by a sports medicine surgeon.

About 17% of surgical conversions were joint replacements, suggesting that the oUCC saw a healthy balance between acute traumatic injuries and more chronic complaints. This notion is further supported by multiple osteoarthritis-related diagnoses among the most commonly encountered during the study period. While the percentage of trauma surgical conversions was less than 1%, this is likely secondary to fracture care diversion to the other subspecialties within our orthopedic practice (i.e., distal radius fractures to hand and wrist surgeons). However, the exceptionally low rate of patients transferred directly to an ED indicates that the overwhelming majority of injuries did not require the immediate care of a traumatologist, and likely such high acuity patients instead went directly to an ED. Overall, the oUCC scope extended considerably beyond simply treating acute injuries to also serving patients who sought care for more chronic pathologies. Therefore, the potential for surgical conversions existed across most subspecialties.

Our study has several limitations, and most are related to its retrospective design and data availability. First, the most common diagnosis codes encountered were broad. It is likely that injuries requiring advanced imaging for discrete diagnosis (such as a meniscal tear, biceps rupture, etc.) were diagnosed as pain or generalized sprain. Also, due to comfort, experience, and/or knowledge, the oUCC providers may not have utilized more specific subspecialty diagnostic codes. Second, during our study period, the coding system changed from ICD-9 to ICD-10, which makes it difficult to compare codes between years. Third, we relied on the divisional assignment of surgical conversions to characterize the types of pathologies requiring surgery. There is certainly some degree of overlap of such assignments within our practice, and this assignment is specific to our practice and may not be generalizable to other practices. For example, a patient with a rotator cuff tear may be treated by either a shoulder and elbow surgeon or sports medicine surgeon. Fourth, it is likely that some patients triaged within the oUCC subsequently sought surgical consultation outside of the practice. Given that these patients elected to come to our oUCC and were scheduled follow-up appointments while in the oUCC, we believe that this occurred relatively infrequently. To the extent that this did occur, however, our surgical conversion rate would be an underestimation. Finally, we were unable to delineate the number of patients seen who were already established in the practice. Providing established patients access to the oUCC for their condition may help with patient retention if they would otherwise have had to wait for an outpatient clinic appointment and potentially would have scheduled with another practice during that time. Also, the oUCC likely saw a number of postoperative patients and added a point of care for urgent postoperative concerns. While we were unable to assess this directly within the scope of our retrospective review, this utility has been previously described with oUCC access shown to decrease the odds of ED utilization within 90 days of total joint arthroplasty [10].

## Conclusions

Despite its limitations, the present study provides data that is currently underreported in the literature regarding the use and diagnoses encountered in a single oUCC. As can be interpreted through existing literature and the currently presented data, oUCCs are an effective means of expanding care to patients and increasing the local footprint and volume of the orthopedic practice. Their value is proven, and likely to expand in response to the COVID-19 pandemic. Most patients treated during the study period did not require emergent ED transfer and a small percentage required surgical intervention for their presenting complaints. Continued monitoring of the type of patients seen within oUCCs will increase efficacy and optimize patient care.
